# MiRNA-145 increases therapeutic sensibility to gemcitabine treatment of pancreatic adenocarcinoma cells

**DOI:** 10.18632/oncotarget.12268

**Published:** 2016-09-27

**Authors:** Yong Lin, Xin Ge, Yiyang Wen, Zhu-Mei Shi, Qiu-Dan Chen, Min Wang, Ling-Zhi Liu, Bing-Hua Jiang, Yuan Lu

**Affiliations:** ^1^ Department of Laboratory Medicine, Huashan Hospital of Fudan University, Shanghai, China; ^2^ The Department of Clinical Laboratory, Central Laboratory, Jing'an District Centre Hospital of Shanghai, Huashan Hospital of Fudan University Jing'An Branch, Shanghai, China; ^3^ State Key Lab of Reproductive Medicine, Key Laboratory of Human Functional Genomics of Jiangsu Province, Jiangsu Key Laboratory of Cancer Biomarkers, Prevention, and Treatment Department of Pathology, Cancer Center, Nanjing Medical University, Nanjing, China; ^4^ Ninggao Personalized Medicine and Technology Innovation Center, Nanjing, China; ^5^ Department of Neurosurgery, The First Affiliated Hospital of Nanjing Medical University, Nanjing, China; ^6^ Department of Pathology, Anatomy and Cell Biology, Thomas Jefferson University, Philadelphia, PA, United States of America

**Keywords:** pancreatic adenocarcinoma, gemcitabine, miR-145, p70S6K1, chemoresistance

## Abstract

Pancreatic adenocarcinoma is one of the most leading causes of cancer-related deaths worldwide. Although recent advances provide various treatment options, pancreatic adenocarcinoma has poor prognosis due to its late diagnosis and ineffective therapeutic multimodality. Gemcitabine is the effective first-line drug in pancreatic adenocarcinoma treatment. However, gemcitabine chemoresistance of pancreatic adenocarcinoma cells has been a major obstacle for limiting its treatment effect. Our study found that p70S6K1 plays an important role in gemcitabine chemoresistance. MiR-145 is a tumor suppressor which directly targets p70S6K1 for inhibiting its expression in pancreatic adenocarcinoma, providing new therapeutic scheme. Our findings revealed a new mechanism underlying gemcitabine chemoresistance in pancreatic adenocarcinoma cells.

## INTRODUCTION

Pancreatic adenocarcinoma is the fourth leading cause of cancer-related death around the world with extremely poor prognosis [1]. The five-year survival rate for pancreatic adenocarcinoma patients is approximately 6%, with a median survival of 4-6 months. Thus, pancreatic adenocarcinoma is often diagnostic after it is well-advanced because of its asymptomatic nature [2]. Less than 15% pancreatic adenocarcinoma patients are suitable for operation at the time of diagnosis, which means chemotherapy has been the major treatment for most of the pancreatic adenocarcinoma patients [3]. Gemcitabine (2'-deoxy-2'-difluorodeoxycytidine), a nucleoside analog, has been confirmed to be the first effective drug in pancreatic adenocarcinoma treatment by inhibiting DNA synthesis and stimulating apoptosis of cancer cells [4]. The gemcitabine-related therapy is the medical treatment scanty of clinically effects for pancreatic adenocarcinoma. In addition, only less than 20% pancreatic adenocarcinoma patients are sensitive to gemcitabine treatment, remaining the major challenge for pancreatic adenocarcinoma treatments [5]. Thus, it will contribute to the development of a novel therapeutic strategy to explore the mechanisms underlying gemcitabine resistance and enhance the efficacy of gemcitabine in pancreatic adenocarcinoma treatment [6].

P70S6K1, one of the most important downstream targets of mTOR, can be activated by the PI3K/PTEN/AKT signaling pathway and functions as a key regulator in various cellular functions such as cell cycle, cell apoptosis and chemoresistance [7]. Given the significant role of p70S6K1 in cellular functions, p70S6K1 is proven to be the multifunctional hallmark in cancer therapy [8]. In addition, recent studies demonstrated that p70S6K1 is involved in nucleotide synthesis via regulating enzymatic activities of carbamoyl phosphate synthetase 2 (CAD) [9, 10], indicating the potential role of p70S6K1 in gemcitabine action.

MicroRNAs are small non-coding regulatory RNAs that have been confirmed to participate in human tumorigenesis by directly targeting tumor related genes [11, 12]. Recent studies in pancreatic adenocarcinoma show indispensable roles of miRNAs in various cellular functions, for example, miRNA-21, miRNA-33a, miRNA-155 and miRNA-218 exhibit important roles in tumor proliferation, invasion, metastasis, and apoptosis [13]. However, only a few miRNAs were identified to be involved in gemcitabine chemoresistance, such as miR-21, miR-181b and miR-17-92 cluster [14–16]. MiRNA-145 has been known as a tumor suppressor which is frequently downregulated in various types of cancer including breast cancer [17], colon cancer [18], prostate cancer [19], bladder cancer [20], and osteosarcomas [21]. Although some studies indicate the oncogenic potential of miR-145 in SW620 cells showing increasing cell proliferation/metabolic activity, miRNA-145 participates in cancer development mostly by targeting significant oncogenes and effectively suppressing their expression in different signal pathways, thus inhibiting cancer cell proliferation, invasion and metastasis or enhancing chemosensitivity [22, 23]. Characterization of global microRNA expression reveals anticancer potential of miR-145 in metastatic colorectal cancer. Early evidence from our lab demonstrated that miR-145 negatively regulated p70S6K1 expression at the posttranscriptional level in colon cancer [18]. Here we demonstrate that miR-145 increases sensitivity of pancreatic adenocarcinoma cells to gemcitabine treatment, providing new insights into the role of miR-145/P70S6K1 in mediating gemcitabine chemosensitivity.

## RESULTS

### Gemcitabine treatment induces miR-145 up-regulation in pancreatic adenocarcinoma cells

**Figure 1:**
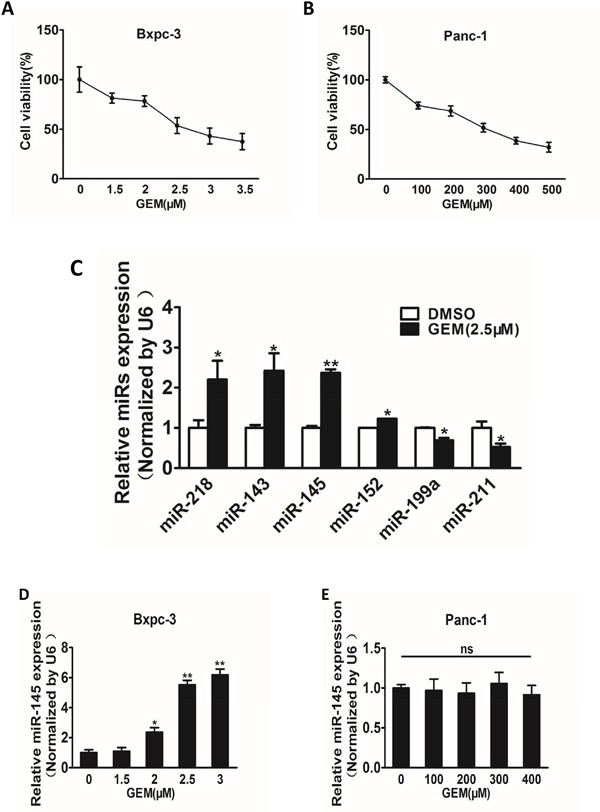
Gemcitabine treatment induces miR-145 up-regulation in gemcitabine-sensitive pancreatic adenocarcinoma cells **A** and **B.** Pancreatic adenocarcinoma cells Bxpc-3 (A) and Panc-1 (B) were treated with gemcitabine (GEM) for 48 h at different doses. The cell viability was analyzed by CCK-8 assay, and normalized to cells without gemcitabine treatment. **C.** Expression levels of miRNAs in Bxpc-3 cells were analyzed by RT-qPCR analysis with the treatment of gemcitabine (2.5 μM) for 48 h. **D, E.** Expression levels of miR-145 in Bxpc-3 (D) and Panc-1 (E) cells treated with different doses of gemcitabine. Data from three independent experiments were shown as mean ± SEM. * indicated significant difference at P<0.05. ;** indicated significant difference at P<0.01.

To identify miRNAs whose expression levels were altered in pancreatic cells in response to gemcitabine treatment, we used CCK8 assay to test the effective concentrations of gemcitabine treatment in Bxpc-3 and Panc-1 cells, and found out that Bxpc-3 was sensitive, whereas Panc-1 was resistant to gemcitabine treatment, with more than 100-fold higher IC50 in Panc-1 cells (Figure 1A and 1B). After determining the appropriate gemcitabine concentrations in these cell lines, we treated Bxpc-3 cells with gemcitabine (2.5 μM) or vehicle control, and quantified miRNA expression profile by using qRT-PCR analysis. We observed that miR-145 was the most significantly up-regulated miRNA upon treatment (Figure 1C), thus we selected miR-145 as a candidate miRNA in tumor progression and chemotherapy for further study. When Bxpc-3 and Panc-1 cells were exposed to gemcitabine treatment at different concentrations, expression levels of miR-145 were increased in a dose-dependent manner in Bxpc-3 cells, whereas there was no significant change on miR-145 expression in Panc-1 cells (Figure 1D and 1E), suggesting that miR-145 expression was substantially up-regulated by gemcitabine treatment in gemcitabine-sensitive cells and the block of miR-145 enhancement in gemcitabine-resistant cells may be a potential mechanism for gemcitabine chemoresistance.

### MiR-145 is involved in cell migration and gemcitabine chemosensitivity in pancreatic adenocarcinoma cells

**Figure 2:**
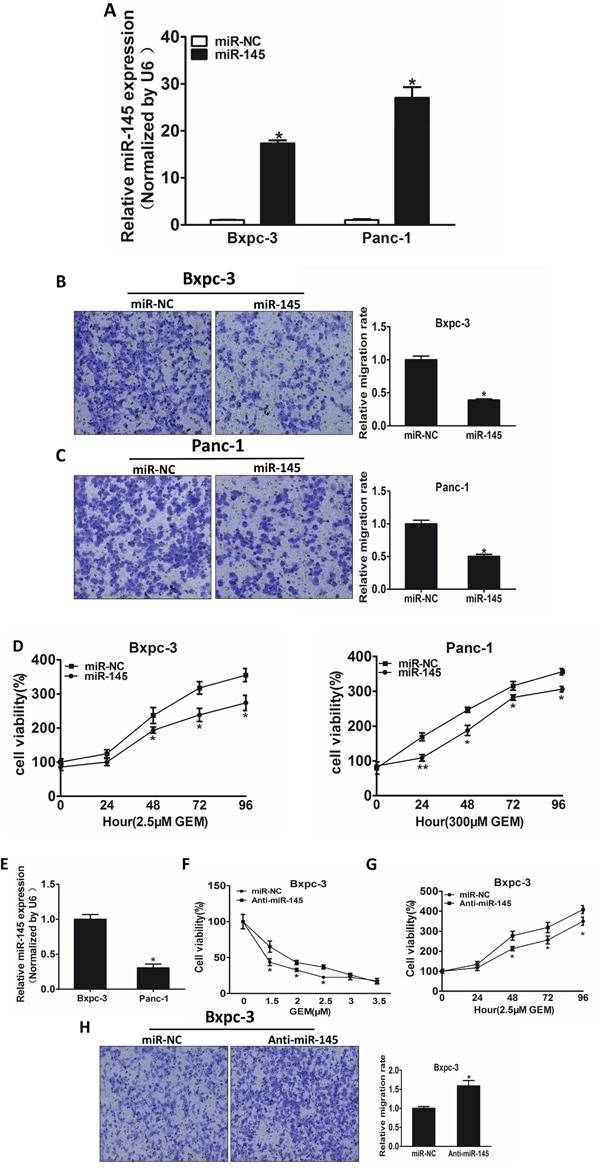
MiR-145 over-expression attenuates cell migration and enhances gemcitabine chemosensitivity in pancreatic adenocarcinoma cells **A.** The levels of miR-145 were detected using RT-qPCR in the two cell lines over-expressing miR-145 or miRNA negative control (miR_NC). **B, C.** Transwell assay was used to measure cell migration in both Bxpc-3 (B) and Panc-1 (C) cells stably expressing negative control miRNA (miR-NC) or miR-145 precursor, and the results were normalized to cells expressing miR-NC. **D.** The effects of miR-145 over-expression on cell viability in Bxpc-3 and Panc-1 cells treated with indicated concentrations of gemcitabine were analyzed using CCK-8 assay. **E.** The endogenous expression levels of miR-145 in Bxpc-3 and Panc-1 cells. **F.** Bxpc-3 and Panc-1 transfected with miR-145 inhibitor (Anti-miR-145) or the negative control miRNA inhibitor (miR-NC) were treated with different concentration of gemcitabine for 48 h. The cell viability was analyzed using CCK-8 assay. **G.** CCK-8 assay was used to analyze the cell viability of Bxpc-3 cells transfected with miR-145 inhibitor or miR-NC for 48h at indicated time points. **H.** Cell migration of Bxpc-3 cells transfected with miR-145 inhibitor. Data represent mean ± SEM from 3 replicates. * indicated significant difference at P<0.05;** indicated significant difference at P<0.01.

Stable cell lines over-expressing miR-145 or negative control miRNA (miR-NC) were established using lentivirus carrying miR-145 or miR-NC, and verified by detecting the expression of miR-145 levels in Bxpc-3 and Panc-1 cells (Figure 2A). One of the key characteristics of malignant tumor is migration, we next investigated the effects of miR-145 over-expression on migration *in vitro*. Forced expression of miR-145 dramatically inhibited 65% and 50% cell migration capacity of Bxpc-3 and Panc-1 cells, respectively (Figure 2B and 2C). The miR-145-overexpressing pancreatic adenocarcinoma cells were used to analyze cell viability upon gemcitabine treatment. The results showed that over-expression of miR-145 in pancreatic adenocarcinoma cells significantly increased the sensitivity to gemcitabine treatment (Figure 2D and 2E). To examine the basal levels of miR-145 expression in these cell lines, we found that compared with Panc-1, Bxpc3 has higher expression levels of miR-145. Therefore, we knocked down miR-145 in Bxpc-3 cells to further verify the biological function of miR-145. We found that miR-145 interference effectively increased the viability of Bxpc-3 cells upon gemcitabine treatment (Figure 2F), and cell proliferation was enhanced by miR-145 inhibition under the treatment of gemcitabine in Bxpc-3 cells (Figure 2G). Furthermore, Transwell assay showed that miR-145 interference enhanced the migration of Bxpc-3 cells.

### P70S6K1 is a direct target of miR-145

**Figure 3:**
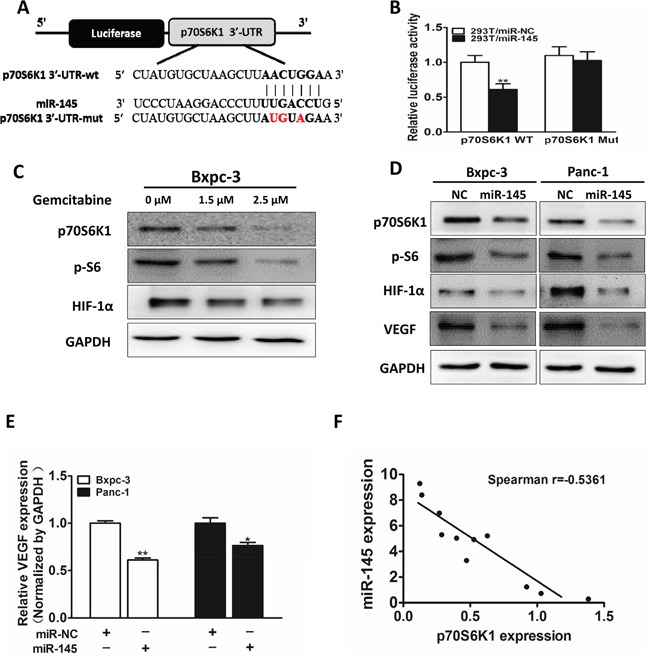
P70S6K1 is a direct target of miR-145 **A.** Schematic diagram of putative miR-145 binding site in the 3′-UTR region of p70S6K1 in human. The seed sequence of miR-145 matches 3'-UTR regions of p70S6K1 (in bold). The mutated nucleotides of the p70S6K1 3'-UTR were labeled in red. **B.** HEK-293T cells were co-transfected with control (miR-NC) or pre-miR-145 mimics along with WT or mutant p70S6K1 luciferase reporter as indicated. Luciferase activities were measured 24 h post transfection using the dual-luciferase reporter assay system. **C.** Cells were treated with different doses of gemcitabine (0, 1.5 and 2.5 μM), the expression levels of p70S6K1, p-S6 and HIF-1α were determined by Western blotting. GAPDH is used as an internal control. **D.** Cells were transfected with miR-145 or miR-NC. After 72 h, the expression levels of p70S6K1, p-S6, HIF-1α and VEGF were determined as above. **E.** VEGF levels were measured by RT-qPCR in cell lines stably overexpressing miR-NC and miR-145, and normalized to level of GAPDH. F: Spearman′s correlation analysis was used to detect the correlation between expression levels of p70S6K1 and miR-145 at mRNA level in human pancreatic tumor specimens. Data represent mean ± SEM of three replicates. * indicated significant difference at P<0.05;** indicated significant difference at P<0.01.

Growing evidence indicates that p70S6K1 pathway is involved in cell growth, metastasis and drug resistance. Bioinformatics program was used to predict targets of miR-145, among which p70S6K1 was one of the putative targets of miR-145. Next, we examined whether p70S6K1 is a direct target of miR-145. The sequences of p70S6K1 3'-UTR region with miR-145 potential binding site was shown in Figure 3A. To verify whether miR-145 directly targets p70S6K1, p70S6K1 3′-UTR sequences containing putative binding sites of wild type (WT) or the mutant one (mut) (Figure 3A) were cloned into pMIR-REPORTER vector. HEK293T cells were co-transfected with the wild type (WT) or mutant (Mut) p70S6K1 3'-UTR luciferase reporter vector together with miR-145 mimics or miR-NC for 24 h, and luciferase activities in those cells were measured. Overexpression of miR-145 significantly inhibited the luciferase activities of wild-type, but not mutant p70S6K1 3'-UTR reporter in HER293T cells (Figure 3B), suggesting that miR-145 targets p70S6K1 through the binding to the seed sequence of its 3'-UTR. To determine whether p70S6K1 was down-regulated under gemcitabine treatment, Bxpc-3 cells were treated with gemcitabine for 48 h and the protein levels of p70S6K1, p-S6, HIF-1α and GAPDH were examined. We found that the levels of p70S6K1, p-S6 and HIF-1α were significantly suppressed in a dose-dependent manner (Figure 3C). In addition, miR-145 over-expression markedly suppressed protein expression levels of p70S6K1, p-S6, HIF-1α and VEGF in pancreatic cancer cells (Figure 3D), confirming that miR-145 targets p70S6K1 and inhibits relative signaling molecules in these cells. Consistent with HIF-1α and VEGF protein down-regulation in miR-145-overexpressing Bxpc-3 and Panc-1 cells, forced expression also suppressed VEGF mRNA levels in these cells (Figure 3E). Furthermore, as shown in Figure 3F, we measured mRNA levels of miR-145 and p70S6K1 in pancreatic adenocarcinoma tumor tissues and determined the correlation between p70S6K1 levels and miR-145 expression levels (Figure 3F). Spearman's rank correlation analysis showed that expression levels of p70S6K1 and miR-145 in pancreatic adenocarcinoma specimens were inversely correlated (Spearman's correlation r = −0.5361).

### Overexpression of p70S6K1 reverses the inhibitory effect of miR-145 on cell migration and chemoresistance

**Figure 4:**
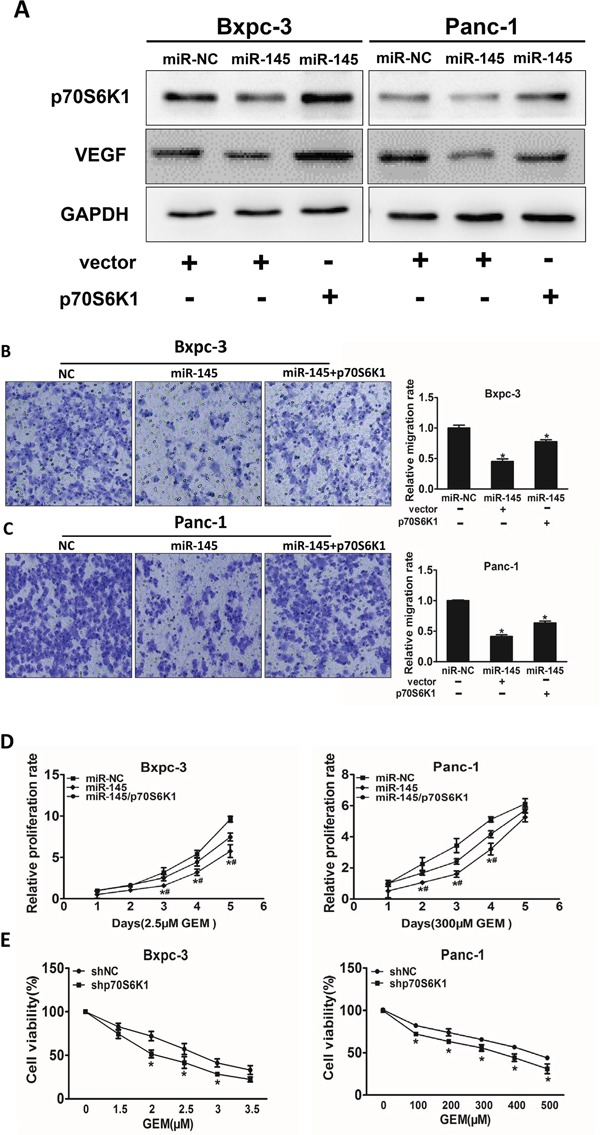
Over-expression of p70S6K1 reverses the inhibitory effects of miR-145 **A.** Bxpc-3 and Panc-1 cells stably expressing miR-NC or miR-145 were transfected with p70S6K1 cDNA construct without 3'-UTR or vector control. After 72 h, p70S6K1 and GAPDH expression levels were measured using immunoblotting assay as above. **B, C.** The effects of p70S6K1 forced expression on cell migration were analyzed using Transwell assay in Bxpc-3 (B) and Panc-1 (C) cells stably expressing miR-NC or miR-145. **D.** The cells above were treated with indicated concentrations of gemcitabine at different time point, cell proliferation was analyzed using CCK8 Assay Kit and normalized to the number of miR-NC group without gemcitabine treatment. **E.** Bxpc-3 and Panc-1 cells were transduced with lentivirus carrying shRNA against p70S6K1 or negative control (shNC) and selected with puromycin. The stable cells were treated with different doses of gemcitabine and cell viability was determined as above. Data represent mean ± SEM. of three replicates. *indicates significant difference compared to control at P<0.05; #indicates significant difference compared to miR-145 treatment alone at P<0.05.

To identify potential molecular mechanism of miR-145 in pancreatic adenocarcinoma development and treatment resistance, we determined whether miR-145 inhibited cell migration and cell viability under gemcitabine treatment though p70S6K1. The overexpression of miR-145 decreased endogenous p70S6K1 protein levels, whereas forced expression of p70S6K1 without 3'-UTR dramatically increased p70S6K1 expression levels (Figure 4A). Over-expression of p70S6K1 rescued the ability of cell migration which was suppressed by miR-145 over-expression in pancreatic adenocarcinoma cell lines (Figure 4B and 4C). Furthermore, cell growth rate in the presence of gemcitabine was assayed by CCK-8 proliferation assay at different time points. Our results showed that the restoration of p70S6K1 expression in the cells stably over-expressing miR-145 significantly increased gemcitabine chemoresistance (Figure 4D). To further study whether p70S6K1 is required for gemcitabine-resistance in pancreatic cancer cells, we confirmed that knockdown of p70S6K1 using its shRNA significantly increased cell viability upon gemcitabine treatment in both cell lines (Figure 4E).

### MiR-145 inhibits VEGF transcriptional activation through targeting p70S6K1 in pancreatic adenocarcinoma cells

**Figure 5:**
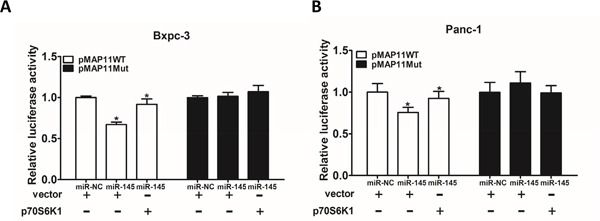
MiR-145 inhibits VEGF transcriptional activation through targeting p70S6K1 in pancreatic adenocarcinoma cells **A.** Bxpc-3 cells were co-transfected with wild-type VEGF reporter (pMAP11-WT) or mutant VEGF reporter (pMAP11-mut), miR-NC negative control or miR-145 precursor, p70S6K1 cDNA or vector control, and pRL-TK plasmid. The luciferase activities were measured using firefly and Renilla dual luciferase reporter assay, and normalized to those of the control. **B.** The similar experiment was performed using PANC-1 cells. Data represent mean ± SEM. from three replicates. * indicated significant difference at P<0.05.

Previous studies have shown the importance of p70S6K1/HIF-1α/VEGF pathway in the regulation of angiogenesis and tumor progression. It has been reported that HIF-1α activates the expression of VEGF gene by binding to the hypoxia response element (HRE) in the VEGF promoter region. To determine whether miR-145 attenuates VEGF transcriptional activation through targeting p70S6K1, we analyzed the effect of miR-145 on the wild type VEGF promoter reporter plasmid (pMAP11WT) containing the HIF-1α binding site or the mutant plasmid (pMAP11Mut) with or without p70S6K1 over-expression. As shown in Figure 5A and 5B, forced expression of p70S6K1 restored miR-145-inhibited the wild type VEGF reporter activity in Bxpc-3 and Panc-1 cells, but did not affect the mutant reporter activity, indicating that miR-145 inhibits VEGF transcriptional activation by targeting p70S6K1.

## DISCUSSION

Pancreatic adenocarcinoma is one of the most common digestive tract malignant tumors, which is difficult in early diagnosis and treatment. In present, distant metastasis, relapse, and drug resistance are still very difficult problems in cancer therapy. Gemcitabine, a first-line treatment drug of pancreatic adenocarcinoma, is widely used in clinical practice. Currently, some literatures suggest that miRNAs can affect drug sensitivity of tumor cells to gemcitabine treatment, including miR-218, miR-33a and miR-223 [24–26], but the role and molecular mechanism of miRNAs in pancreatic adenocarcinoma cells to gemcitabine treatment have not been reported. We found that compared with Bxpc-3, a much higher concentration of gemcitabine was required to inhibit the cell viability of Panc-1, confirming that Panc-1 is a gemcitabine-resistant cell line. We also found that gemcitabine could up-regulate expression levels of miR-145 in Bxpc-3, the gemcitabine-sensitive cells, but not in Panc-1 cells, suggesting specific role of miR-145 in gemcitabine chemosensitivity in pancreatic adenocarcinoma cells.

MiR-145 is known as a tumor suppressor, which plays an important role in tumor development. The expression levels of miR-145 are found to be down-regulated in many types of cancers, including pancreatic adenocarcinoma [27–31]. The expression profiles and biological role of miR-145 in pancreatic adenocarcinoma has been reported [31, 32], but the role and mechanisms of miR-145 in regulating drug resistance remain to be elucidated. In this study, we found that in both gemcitabine-sensitive cells Bxpc-3 and gemcitabine-resistant cells Panc-1, overexpression of miR-145 significantly inhibited cell migration by directly targeting p70S6K1. Moreover, overexpression of miR-145 increased chemosensitivity of cancer cells to gemcitabine treatment *in vitro*, indicated by decreased cell viability. Furthermore, p70S6K1 is a direct target of miR-145 in pancreatic cancer cells. Forced expression of p70S6K1 reversed miR-145-suprressed cell proliferation and drug resistance of pancreatic adenocarcinoma cells, suggesting that p70S6K1 plays a central role in miR-145-induced chemosensitivity to gemcitabine treatment. MiR-143 may have similar effect and regulation mechanism as miR-145 in cells, which would also be interesting to be studied in the future.

The activation of p70S6K1 affects the expression levels of two important angiogenesis-related downstream signaling molecules, HIF-1α and VEGF, which are involved in tumorigenesis and cancer development [33–37]. It has also been demonstrated that HIF-1α regulates VEGF expression at the transcriptional level by binding to its promoter [38]. In our study, overexpression of miR-145 suppressed the expression levels of HIF-1α and VEGF, which is consistent with its inhibitory effect on p70S6K1. Furthermore, forced expression of p70S6K1 in miR-145-over-expressing pancreatic adenocarcinoma cells restored the expression levels of HIF-1α and VEGF which were inhibited by miR-145, suggesting that miR-145 controls expression of HIF-1a and VEGF by targeting p70S6K1 in the cells.

In summary, our findings showed that Panc-1 resistance to gemcitabine is due to the down-regulation of miR-145, suggesting that miR-145 may be an indicator for the development of pancreatic adenocarcinoma. Forced expression of miR-145 inhibited the cell viability, migration, HIF-1α, VEGF expression and enhanced the chemosensitivity to gemcitabine through its direct target p70S6K1 in pancreatic adenocarcinoma. These results suggest that miR-145 may be a potential target for adjuvant therapy to enhance the chemosensitivity to gemcitabine in the future. Our findings provided a new molecular basis for the application of miR-145 in pancreatic adenocarcinoma, which may be helpful in developing microRNA-based drug for treating pancreatic adenocarcinoma in the future.

## MATERIALS AND METHODS

### Cell culture and reagents

Human pancreatic adenocarcinoma cell lines, Panc-1 and Bxpc-3 were maintained in Dulbecco's Modified Eagle's medium with high glucose and RPMI-1640 medium with low glucose, respectively, and supplemented with 10% fetal bovine serum and antibiotics (100 units/ml penicillin and 100 mg/ml streptomycin). Cells were incubated in a 5% CO2 incubator at 37°C. Trypsin (0.25%) solution was used to detach the cells from the culture flask. P70S6K1 and VEGF antibodies were purchased from Santa Cruz Biotechnology (Santa Cruz, CA, USA). Antibodies against HIF-1α and GAPDH were purchased from Bioworld Technology (Atlanta, Georgia, USA). Gemcitabine was obtained from Sigma (St. Louise, MO).

### Luciferase reporter assays

The 3'-UTR of p70S6K1 constructs containing predicted miR-145 seed-matching sites from cDNA library were amplified by PCR using Pfu DNA polymerase, then cloned into pMIR-REPORTER vector (Ambion). Primers of wide type (WT) and corresponding mutant sites (Mut) used for reporter constructions were shown in Supplementary Table S1. All the 3'-UTR products were sequenced and verified. For reporter assays, HEK-293T cells were transiently cotransfected with WT or Mut plasmid, pRL-TK plasmid and miRNA-145 mimics (GenePharma, Shanghai, China) or miRNA-negative control (miR-NC) using lipofectamine 2000 (Invitrogen). Firefly and renilla luciferase activities were measured 24 h after transfection using a dual luciferase assay kit (Promega, Shanghai, China). The relative luciferase activities were calculated and normalized to the control.

To detect the effects of miR-145 on transcriptional activation of VEGF, the downstream of p70S6K1, VEGF reporter plasmid pMAP11-WT or pMAP11-Mut was co-transfected into Bxpc-3 and Panc-1 cells with pRL-TK plasmid and miR-145 mimics or miR-NC. Firefly and Renilla luciferase activities were measured 24 h after the transfection. Experiments were performed in three independent replicates.

### RNA extractions, reverse transcription PCR and quantitative real time- PCR

Total RNAs were extracted from cells with TRIzol (Invitrogen, CA, USA). The stem-loop RT-PCR assay was performed to quantify the miRNAs expression levels using the HiScript RT Reagent Kit (Vazyme Biotech, Nanjing, China) according to the manufacturer's instructions. To determine the mRNA levels of VEGF, total RNAs were reversely transcribed using HiScript RT Reagent Kit (Vazyme Biotech, Nanjing, China). RT-qPCR was performed to detect mRNA expression levels using the SYBR Premix DimerEraser (Takara, Dalian, China) on a 7900HT system. The expression levels of U6 or GAPDH were used as internal control. The expression levels of miRNAs in each group were calculated by relative quantification (2-^ΔΔCt^). The primers used in this study are shown in Supplementary Table S1.

### Cell proliferation

To detect the gemcitabine effects on the pancreatic adenocarcinoma cell viability, cells were seeded into 96-well plates at 2000 per well. After cellular adhesion, medium containing gemcitabine at different concentrations was added to the cells. Cell viability was assessed using the CCK8 Assay (CCK8 kit, Dojindo Laboratories, Kumamoto, Japan) 48 h after the transfection. CCK8 reagents were prepared in fresh medium (100 μl medium containing 10 μl CCK8 solutions) and applied to the cells. The absorbance at 450 nm for each well was analyzed on a spectrophotometer.

To determine the effects of miR-145 on cell growth, Bxpc-3 and Panc-1 cells were seeded in a 6-well plate and cultured overnight. After transient transfection with miR-145 and miR-NC precursors, the cells were trypsinized and seeded into a 96-well plate. CCK8 Assay was used to measure cell proliferation at different indicated time points.

### Cell migration assay

Transwell chambers (24-well insert; Corning, NY, USA) were used to analyze cell migration in accordance with the manufacturer's instructions. Cells were seeded in the upper well of the chamber in DMEM or RPMI-1640 medium without serum, while the lower chamber well contained DMEM or RPMI-1640 medium supplemented with 10% FBS to stimulate cell migration. After incubation for 12 h, cells on the upper membrane surface were removed while the bottom cells were fixed with 3% paraformaldehyde, stained with 0.1% crystal violet, and 5-6 random fields were collected for quantification for each well. The cells were finally extracted with 33% acetic acid and detected quantitatively using a standard microplate reader (at 570 nm). Three independent experiments were performed for each experimental group.

### Establishment of stable cell lines expressing miR-145

Lentivirus carrying miR-145 and miR-NC were packaged using lentiviral packaging kit in HEK-293T cells according to the manufacturer's instructions (Open Biosystems, AL, USA). Stable Bxpc-3 and Panc-1 cells over-expressing miR-145 or miR-NC were generated by the infection of lentivirus (Open Biosystems), followed by puromycin (Life Technologies) selection.

### Western blotting

Cells were lysed with RIPA buffer (150 mM NaCl, 100 mM Tris, pH 8.0, 0.1% SDS, 1% Triton X-100, 1% sodium deoxycholate, 5 mM EDTA, and 10 mM NaF) supplemented with 1 mM sodium vanadate, 2 mM leupeptin, 2 mM aprotinin, 1 mM phenylmethylsulfonyl fluoride (PMSF), 1 mM DTT, and 2 mM pepstatin. Total proteins were collected by centrifugation at 12,000 rpm 4°C for 15 min. After quantification, 20 μg protein lysates were separated by SDS–PAGE and subsequently transferred to polyvinylidene difluoride membranes (Millipore, Billerica, MA, USA), membranes were blocked with 5% nonfat dry milk for 2 h and incubated with primary antibodies. Protein bands were detected by incubation with horseradish peroxidase-conjugated antibodies and visualized with an electrochemiluminescence detection system (Thermo Scientific).

### Statistical analysis

Values were obtained from at least three independent experiments and presented as means ± SEM. Comparison between two groups was conducted using the t-test, while comparisons between multiple groups of data were performed using analysis of variance (ANOVA). It was considered to be statistically significant at P<0.05.

## SUPPLEMENTARY TABLE


