# Supplementary material for: MiRNA-145 increases therapeutic sensibility to gemcitabine treatment of pancreatic adenocarcinoma cells
Source: Oncotarget. 2016 Sep 27;7(43):70857–68. doi: 10.18632/oncotarget.12268 (PMC5342594; doi:10.18632/oncotarget.12268)
Supplement: Supplementary file 1 [file oncotarget-07-70857-s001.pdf]

MiRNA-145 increases therapeutic sensibility to gemcitabine treatment of pancreatic adenocarcinoma cells

SUPPLEMENTARY TABLE

Supplementary Table S1: Primers used in this study

Primer name	Sequence 5'-3'
miR-145 RT	CTCAACTGGTGTCGTGGAGTCGGCAATTCAGTTGAGAGGGATTC
miR-145-F	ACACTCCAGCTGGGGTCCAGTTTTCCCAGGA
miR-145-R	TGGTGTCGTGGAGTCG
U6 RT	AACGCTTCACGAATTTGCGT
U6-F	CTCGCTTCGGCAGCACA
U6-R	TGGTGTCGTGGAGTCG
miR-218 RT	GGTCGAACGCCTAACGTCACATGGTTAG
miR-218-F	CGAGTGCATTTGTGCTTGATCTA
miR-218-R	TAATGGTCGAACGCCTAACGTC
miR-143 RT	TCGTCGAGATAAGCTGTGTGTGAGCTACA
miR-143-F	GTTCGCTGAGATGAAGCACTG
miR-143-R	GCTCGTCGAGATAAGCTGTGTG
miR-152 RT	GTCGTATCCAGTGCAGGGTCCGAGGTATTCGCACTGGATACGACCCAAGT
miR-152-F	GTGCAGGGTCCGAGGT
miR-152-R	TGCAGGGTCCGAGGTATTC
miR-199a RT	AAGGCGATTGATACGAGTCAGAACAGGTA
miR-199a -F	GGTCTCCCCAGTGTTCAGACTA
miR-199a -R	AGAAGGCGATTGATACGAGTCA
miR-211 RT	GTCGTATCCAGTGCAGGGTCCGAGGTATTCGCACTGGATACGACAGGCGA
miR-211-F	CTGCTTGGACCTGTGACCTGT
miR-211-R	TGCAGGGTCCGAGGTATTC
WT-p70-F	CGGGGATCCGGGTGGACCTGGGGTTTATTT
WT-p70-R	CCCCTCGAGTTCATCAAAAGGCCATCAAAT
MT-p70-F	GCAGTACTGCTATGTGCTAAGCTTAACTCCAAGCCTTGGAATGGG
MT-p70-R	CCCATTCCAAGGCTTGGAGTTAAGCTTAGCACATAGTACTG
VEGF-F	CGAGGGCCTGGAGTGTG
VEGF-R	CCGCATAATCTGCATGGTGAT
GAPDH-F	ATGGGTGTGAACCATGAGAAGTATG
GAPDH-R	GGTGCAGGAGGCATTGCT
